# Pretrained transformer framework on pediatric claims data for population specific tasks

**DOI:** 10.1038/s41598-022-07545-1

**Published:** 2022-03-07

**Authors:** Xianlong Zeng, Simon L. Linwood, Chang Liu

**Affiliations:** 1 Electrical Engineering and Computer Science, Ohio University, Athens, 45701 USA; 2 Research Information Solutions and Innovation, Nationwide Children’s Hospital, Columbus, OH 43205 USA

**Keywords:** Computational biology and bioinformatics, Health care

## Abstract

The adoption of electronic health records (EHR) has become universal during the past decade, which has afforded in-depth data-based research. By learning from the large amount of healthcare data, various data-driven models have been built to predict future events for different medical tasks, such as auto diagnosis and heart-attack prediction. Although EHR is abundant, the population that satisfies specific criteria for learning population-specific tasks is scarce, making it challenging to train data-hungry deep learning models. This study presents the Claim Pre-Training (Claim-PT) framework, a generic pre-training model that first trains on the entire pediatric claims dataset, followed by a discriminative fine-tuning on each population-specific task. The semantic meaning of medical events can be captured in the pre-training stage, and the effective knowledge transfer is completed through the task-aware fine-tuning stage. The fine-tuning process requires minimal parameter modification without changing the model architecture, which mitigates the data scarcity issue and helps train the deep learning model adequately on small patient cohorts. We conducted experiments on a real-world pediatric dataset with more than one million patient records. Experimental results on two downstream tasks demonstrated the effectiveness of our method: our general task-agnostic pre-training framework outperformed tailored task-specific models, achieving more than 10% higher in model performance as compared to baselines. In addition, our framework showed a potential to transfer learned knowledge from one institution to another, which may pave the way for future healthcare model pre-training across institutions.

## Introduction

The rapid growth of electronic health records (EHR) promotes the use of data-driven modeling to improve care delivery and care management for individual patients. Specifically, novel applications are emerging that use state-of-the-art deep learning approaches such as recurrent neural networks^1^, convolutional neural networks^2^ and transformers^3^, to predict future medical events. With sufficient patient samples, these deep learning models can progressively extract relevant features and show promising model performance for various predictive tasks. For example, doctorAI, which yields promising results for predicting subsequent medical visits’ diagnosis and medication, was trained on a dataset with more than 200,000 patient records.

One prerequisite for training most deep learning models is the availability of substantial amounts of high-quality data^4,5^. Although the large-scale EHR database contains millions of patient records, these records are often not entirely applicable for various reasons, such as a limited number of cases for rare conditions and diseases^6^, restricted access to the entire database due to privacy concerns^7^, difficulty in data cleaning and merging^8^ (especially if collected from different institutions^9^). These limitations hinder the data acquisition process and, therefore, restrict the opportunities to develop data-hungry deep learning models, which may slow down the computational advances in healthcare and impede the improvement in care delivery.

Various methods have been proposed to address the data insufficiency issue, including synthetic data generation and incorporating medical domain knowledge. For example, Lee et al.^10^ proposed an autoencoder-based deep generative model to learn and synthesize realistic sequential EHR data; Buczak et al.^11^ generate EHR records by utilizing domain-specific knowledge and actual data; Ma et al.^12^ proposed the PRIME model to leverage medical knowledge graph for rare medical concepts. However, these existing methods share the following drawback: they do not utilize the large amounts of “non-qualified” EHR records that are excluded during the patient cohort selection process.

Another approach to tackle the data scarcity problem is transfer learning (also known as model pre-training), which aims to learn good representations in an unsupervised manner to boost model performance in the downstream tasks. Transfer learning has been proven effective in a wide range of Natural Language Processing (NLP) tasks^13–16^ and Computer Vision (CV) tasks^17–19^. Recent studies also applied transfer learning techniques to the healthcare domain. For example, Li et al.^20^ proposed BEHRT to pre-train the Masked Language Model (MLM) on more than one million patient records to capture the semantic meaning of medical codes, BioBERT^21^ and Clinical-BERT^22^ pre-trained BERT (Bidirectional Encoder Representations from Transformers)^23^ on clinical text for clinical NLP tasks. Despite their promising results, most of these studies focus on pre-training models on free-text; therefore, model pre-training on structural claims data, especially pediatric claims data, are neglected.

Claims data, a special kind of EHR, which is mostly used for financial purposes, contains rich clinical information. Such information can reflect the disease progression of patients and offers valuable support for healthcare analysis. Various data-driven models built for different predictive tasks are based on claims data, and, therefore, it makes computational sense to adapt the pre-training framework on claims data. However, pre-training models on claims data have been minimally explored and face unique challenges. First, medical visits are unevenly distributed. The time span between two consecutive medical visits is likely to differ. Second, medical claims contain different types of variables, such as medical codes, claim type, service date, and expenditure. These variables contain clinical meaningful information and need to be modeled collectively. Finally, some rare medical codes, such as brain cancer (ICD-9 191.9), have low frequency even in a large healthcare database and, therefore, suffer from severe sparsity.

Our goal is to learn a universal representation of claims data that can transfer knowledge with little adaptation to a wide range of population-specific tasks. This study took a semi-supervised approach for predictive healthcare tasks using a combination of unsupervised pre-training and supervised fine-tuning. Specifically, we proposed Claim Pre-Training (Claim-PT), a two-stage framework that first uses Next Visit Prediction (NVP) and Categorial Prediction (CP) as objectives to pre-train the initial parameters of the transformer-based neural network. The NVP predictive objective helps capture the relationships between medical claims and the co-occurrence information of medical codes, while the CP predictive objective induces medical domain knowledge to mitigate the rare medical code sparsity issue. Next, the pre-trained model is adapted to a population-specific predictive task, such as asthma exacerbation prediction, using the corresponding selected patient cohort.

We pre-train our model on a large-scale pediatric claims dataset containing more than one million unique patients. We evaluate our framework on two population-specific predictive tasks (i.e., suicide risk prediction and asthma exacerbation prediction) and compare the model performance with discriminatively trained models. Experimental results show that our model can learn generalized patient representation through encoding the sequential medical claims and significantly outperforms baselines. We also evaluate our model’s transfer learning ability across institutions. The empirical results confirm that our framework possesses great potential for transfer learning across different healthcare organizations, indicating that organizations with insufficient pediatric claims data can also benefit from our pre-trained model.

We publicly release the pre-trained model along with the population-specific preprocessing steps for claims data on GitHub: https://github.com/drxzeng/Claim-PT. In summary, we make the following contributions:

- We train and publicly release Claim-PT, a transformer-based framework trained on a large real-world pediatric claims database. To the best of our knowledge, Claim-PT is the first pre-trained and fine-tuned framework on pediatric claims data, that can deliver a significant performance boost for population-specific predictive tasks.
- We demonstrate that our framework can utilize the general claims records for medical knowledge understanding. The pre-trained framework helps to improve downstream population-specific medical predictive tasks and outperforms tailored task-specific baselines. In addition, we show that the pre-trained framework has great potential at knowledge generalization across institutions, paving the way for future care coordination and delivery between healthcare organizations.

## Related work

In this section, we first review the related research for transfer learning using claims data. Specifically, we focus on deep learning approaches. Next, we present several medical tasks using claims data and the corresponding predictive models.

### Transfer learning using claims data

Transfer learning is an approach where deep learning models are first trained on a large (unlabeled) dataset to learn generalized parameter initialization and perform similar tasks on another dataset. Several state-of-the-art results in the NLP and CV domain are based on transfer learning solutions^24,25^.

Recently, researchers applied transfer learning techniques to the medical domain. Transfer learning enables deep learning models to capture comprehensive contextual semantics, which can benefit the downstream predictive tasks. For example, MED-BERT^26^ pre-trained contextualized medical code embeddings on large-scale claims data and illustrate that the pre-trained embeddings can improve model performance on the downstream tasks. Med2vec, proposed by Choi et al.^27^ is a skip-gram-based model that can capture the co-occurrence information between medical visits. Med2vec is able to learn semantic meaningful and interpretable medical code embedding, which can benefit the predictive tasks and provide clinical interpretation. BioBERT^21^, is a pre-trained biomedical language model trained on biomedical text instead of claims data, aims at adapting the language model for biomedical corpora.

These studies demonstrate the effectiveness of the pre-train and fine-tune framework with respect to boosting model performance on the downstream predictive tasks, especially when the data size is limited. However, none of the previous research focuses on pediatric claims data. We want to explore whether the pre-train and fine-tune paradigm on pediatric claims can benefit downstream predictive tasks, specifically with a population-specific patient cohort.

### Predictive models using claims data

There has been active research in modeling the longitudinal claims data for various predictive tasks. Generally, these studies can be divided into two groups: works that focus on predicting a specific future medical event, such as suicide risk prediction, asthma exacerbation prediction; and works that focus on a broader range of medical events, such as auto diagnosis and chronic disease progression modeling.

Various deep learning models have been proposed to model claims data for a specific future medical event prediction. Su et al.^28^ proposed a logistic regression model with carefully selected features to predict the suicide risk among children. Xiang et al.^29^ predict the risk of asthma exacerbations and explore the potential risk factors involved in the progression of asthma via a time-sensitive attentive neural network. Zeng et al.^30^ developed a multi-view framework to predict the future medical expenses for better care delivery and care management. Choi et al.^31^ proposed RETAIN to estimate the future heart failure rate with explainable risk factors. For general-purpose disease progressing models, Zeng et al.^3^ proposed a hierarchical transformer-based deep learning model to forecast future medical events. Ma et al.^32^ leverage medical domain knowledge to model the sequential medical codes for the next visit medical code prediction.

One of the main challenges in developing these models is the size of the dataset. The datasets used in previous studies usually contain over a hundred thousand patients, which is large enough to train most deep learning networks. However, for many population-specific predictive tasks or institutions without a large data corpus, training a complex deep learning model from scratch is not feasible and therefore requires transfer learning or alternative techniques.

## Framework

In this section, we first describe the motivation of our study under the real-world scenario. Then we describe the formal definition of the problem of pre-training in healthcare using claims data. Finally, we illustrate our proposed methods in detail.

### Motivation and training procedures

**Figure 1:**
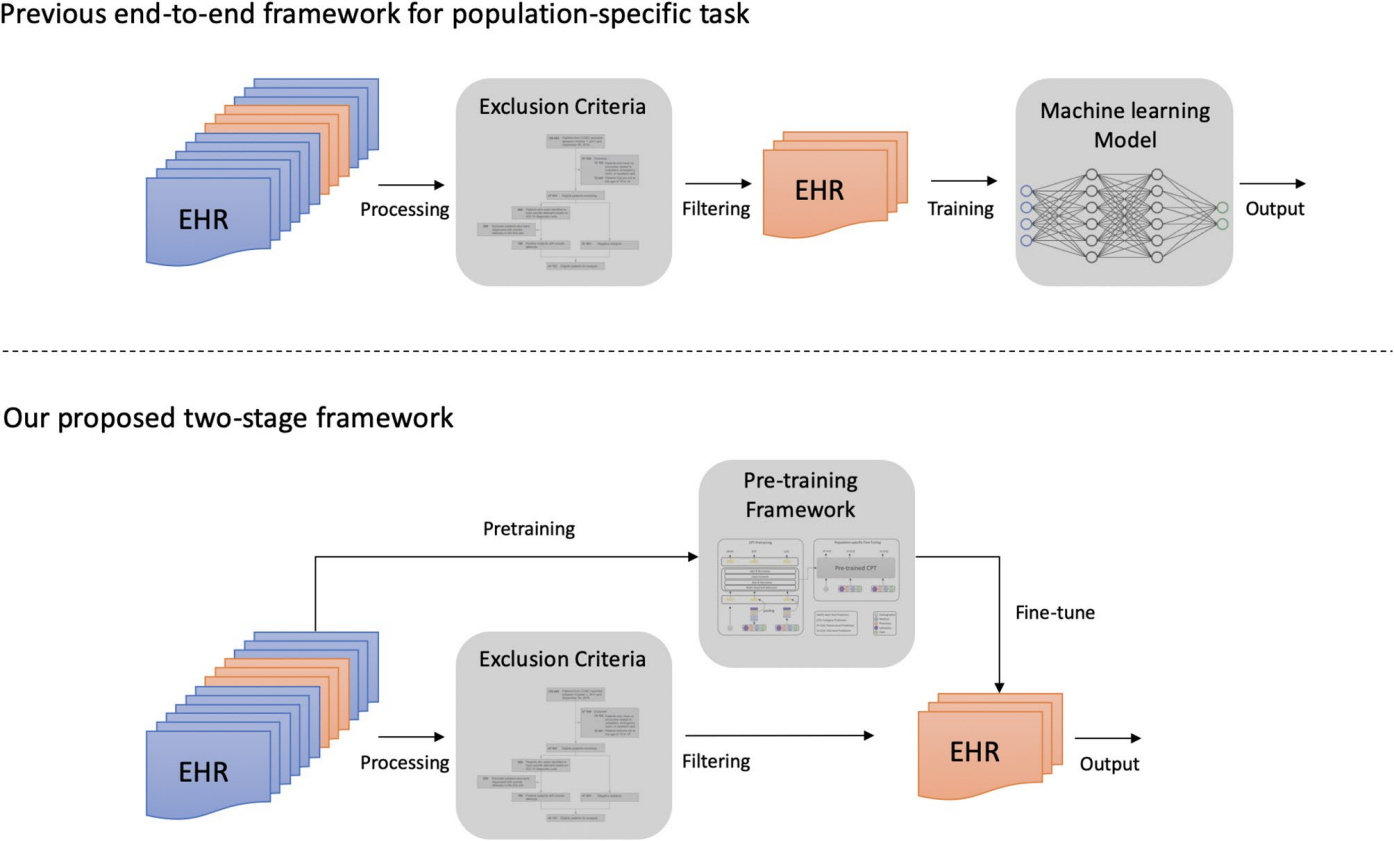
The motivation of our study. The traditional pipeline (top) of most medical predictive tasks requires rigorous criteria to construct the case and control patient cohort, undermining deep learning models’ predictive power and leading to suboptimal performance. Our proposed pre-training and fine-tuning framework (bottom) leverage the excluded patient records and significantly boost the model performance.

Before the formal description of our framework, we would like to explain its underlying motivation and practical scenario by a high-level illustration in Fig. 1. Figure 1 (top) shows the traditional pipeline of building deep learning models on a predictive task in the medical domain. As one can see, numerous claims records are removed during the cohort selection process following the inclusion and exclusion criteria. After the cohort selection process, only patients with specific diseases or satisfy certain criteria can remain as cases or controls, leaving a relatively small data cohort compared to the original database. The small data size makes it difficult to adequately train a deep learning model and leads to suboptimal model performance.

The observation above provides the main intuition and motivation behind our method. In other words, we aim to leverage the patient records that are excluded in the cohort selection process to boost the model performance on population-specific predictive tasks. We achieve this goal through a two-stage pipeline. The first stage is to learn a high-capacity patient representation learning model, i.e., capturing the clinical meaning of medical codes and the temporal information between medical visits. The first stage is complete through training the model on all available patient records. Next, a fine-tuning stage is followed, where we adapt the pre-trained model to a population-specific medical task. In the situation where the selected patient cohort is small, the pre-trained model can transfer valuable medical information from the excluded patient records.

### Unsupervised pre-training

Given a patient in the claims data represented as a sequence of medical visits $v_1, v_2, \ldots , v_i$ ordered by service date *t*. The i-th medical visit $v_i$ contains a set of medical codes $\{c_1, c_2, \ldots , c_j\}\subseteq |C|$ , where |*C*| refers to the vocabulary size of the medical codes. There are three different types of medical visits, i.e., inpatient visit *IP*, outpatient visit *OP*, and pharmacy visit *RX*.

**NVP objective function**. We first use a modified language modeling objective, named Next Visit Prediction (NVP), to maximize the likelihood of the medical codes in the next visit, as shown below:

1 $$\begin{aligned} L_{NVP}(v_i) =\sum _i log P(v_i | v_1 v_2, \ldots , v_{i-1};\theta ), \end{aligned}$$

where *P* is the conditional probability modeled by a deep neural network with parameters $\theta$, and softmax function is adopted to predicts the medical codes of the next visit:

2 $$\begin{aligned} P(v_i | v_1 v_2, \ldots , v_{i-1}) = \dfrac{exp(W_n v_i + b_n)}{\sum _j exp(W_n[j, :]v_i + b_n[j]) } \end{aligned}$$

**CP objective function**. We design another objective function, named Categorial Prediction (CP), aim to mitigate the sparsity issue of rare medical codes:

3 $$\begin{aligned} L_{CP}(v_1, v_2, \ldots , v_{i}) =log P( {\hat{v}}_1, {\hat{v}}_2, \ldots , {\hat{v}}_{i} | v_1, v_2, \ldots , v_{i};\theta ), \end{aligned}$$

where ${\hat{v}}_i$ contains the categorical medical codes, and *P* is the conditional probability modeled by the same deep neural network as in the previous NVP task:

4 $$\begin{aligned} P( {\hat{v}}_1, {\hat{v}}_2, \ldots , {\hat{v}}_{i} | v_1, v_2, \ldots , v_{i})=\sum _i P({\hat{v}}_{i} | v_{i}) \end{aligned}$$

The second objective guide the model to learn visit representation $v_i$ that are representative of the corresponding code categories. The CP task injects the ontology knowledge of medical codes into the embedding process and force the visit encoder to extract effective information that could boost the pre-training model performance. Sigmoid function is adopted to predicts the corresponding category of the each visit based on the medical code:

5 $$\begin{aligned} P({\hat{v}}_{i} | v_{i})= \dfrac{1}{1+e^{-(w_c v_i + b_c)}} \end{aligned}$$

The definition of the category is the medical grouper ID of the medical codes. For example, ICD-9 code 493.00 can be categorized as CCS 28 using the Clinical Classification Software (CCS). In our study, we use CCS as the category grouper for diagnosis codes and procedure code, and NDC Directory from the Food and Drug Administration (FDA) category grouper for medication codes. Correctly capturing the categories for each medical code within the visits helps to induce expert knowledge and is one of the basis of all downstream prediction tasks.

In our experiments, we adopt the state-of-the-art transformer layer as the building block. The medical codes (i.e., diagnoses, procedures and medications), visit types and service date are first map to latent space via embedding matrices. A max pooling layer is then applied to extract the most salient features within a visit. Next, the transformer block, including a multi-headed self-attention operation, position-wise feedforward layer, and skip-connect normalization layer, is applied to produce an output distribution over the target medical visit. In order to generate patient-level embedding, we also include the demographic information as the first token of every patient sequence using the one-hot encoding technique. The hidden state of this token is used as the aggregate patient representation for patient-level classification tasks, while the hidden state of the sequential visits hidden state is used for visit-level classification tasks. The equations are shown below :

6 $$\begin{aligned} E_t= & {} [E_{diag};E_{proc};E_{drug};E_{util};E_{date}] \end{aligned}$$

7 $$\begin{aligned} e_t= & {} maxpool(E_t) \end{aligned}$$

8 $$\begin{aligned} pe,{\hat{e}}_1,{\hat{e}}_2,\ldots ,{\hat{e}}_t= & {} TransLayer([E_{demo};e_1,e_2,\ldots ,e_t]), \end{aligned}$$

where $E_{diag};E_{proc};E_{drug};E_{util};E_{date};E_{demo}$ are the embedding vectors of diagnoses, procedures, medication, visit type, service date and age & gender. *pe* is the hidden state for patient embedding and $e_1,e_2,\ldots ,e_t$ are the hidden state for visit embeddings.

### Population-specific fine-tuning

After training the framework with the objective functions as mentioned above, we adapt the parameters to the downstream population-specific predictive task. We assume a population-specific claims dataset that contains both positive subjects (y = 1) and negative subjects (y = $-1$) . The input medical visit sequences and demographic vector are passed through the pre-trained model to obtain the final transformer block’s activation $pe,{\hat{e}}_1,{\hat{e}}_2,\ldots ,{\hat{e}}_t$. For patient-level classification task, *pe* is then fed into an added linear output layer with parameters $W_{pe}$ to predict y:

9 $$\begin{aligned} P(y | v_1, v_2, \ldots , v_i)=softmax(pe * W_{pe}) \end{aligned}$$

**Figure 2:**
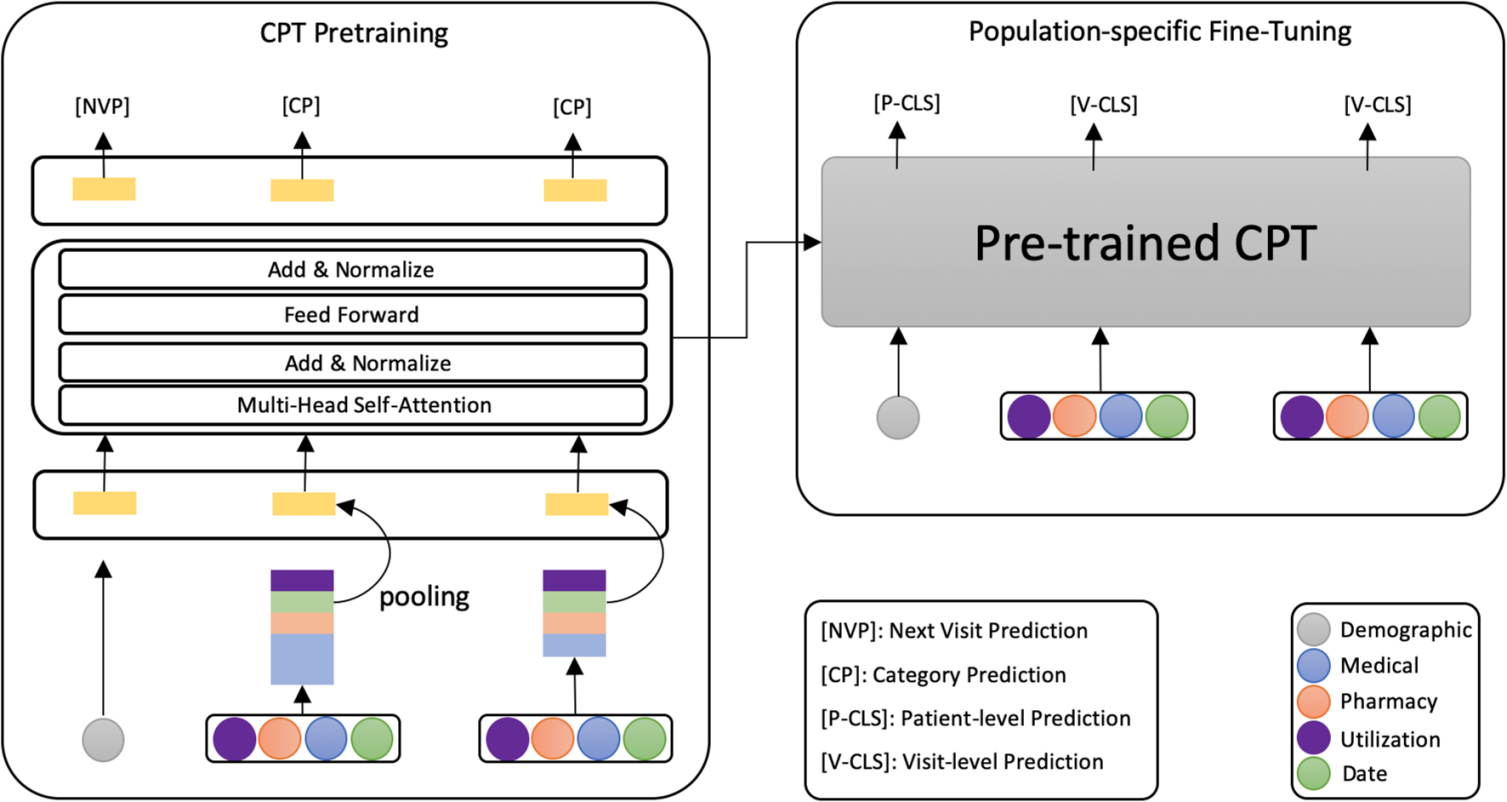
The overview of our pre-training and fine-tuning framework.

Figure 2 illustrate the architecture of our framework. Overall, the only extra parameters we require to train during the fine-tuning stage are $W_{pe}$, and thus we do not need a large patient cohort to train the complex deep learning models from scratch. As a result, our pre-training and fine-tuning framework are suitable for various population-specific predictive tasks.

### Data description

The datasets we used in the experiments are the Partner for Kids (PFK) claims data (for model pre-training), suicide claims (for suicide prediction), asthma claims (for asthma exacerbation prediction), PFK-2013 and MIMIC-3 (for knowledge transfer validation).

#### PFK claims

PFK is one of the largest pediatric care organization for Medicaid in Ohio. Our PFK claims data contains more than 600,000 enrollees’ medical claims from 2010 to 2017. In accordance with the Common Rule (45 CFR 46.102[f]) and the policies of Nationwide Children’s Institutional Review Board, The PFK dataset used in this study is considered a limited dataset and was not considered human subjects research and thus not subject to institutional review board approval.

#### Suicide claims

The suicide dataset consists of Medicaid claims over the years 2013 and 2014, corresponding to patients who have two years of continuous eligibility. Patients diagnosed with suicide attempts are labeled as positive cases (i.e., Medicaid members who have the suicide-related ICD-9 diagnosis codes in their claims), while others are labeled negative. It contains data corresponding to 79,350 patients with 927,318 visits.

#### Asthma claims

The asthma dataset consists of Medicaid claims over the years 2013 and 2014, corresponding to patients who have been diagnosed with asthma (ICD-9 codes 493.XX or ICD-10 codes J45.XX). A total of 22,862 patients with 432,472 visits.

#### PFK-2013

Ten thousand PFK patients’ claims from Jan 2013 to Dec 2013 are first selected to conduct the experiments. The PFK-2013 is extracted from the PFK dataset (but excluded during the pre-training procedure) to mimic the pediatric claims data from another institution.

#### MIMIC-3

A different EHR dataset, MIMIC-3, is used for another across institution experiment. MIMIC-3 is a publicly available clinical dataset that contains ICU patient records for over seven years of observation. This dataset is very different from the PFK pediatric claims data in that it consists of demographically and diagnostically different patients. For example, the median age is 65 in MIMIC-3, while 10 in PFK. The mortality rate among MIMIC-3 patients is about 10%, while close to zero in PFK. Despite the substantial clinical difference between MIMIC-3 and PFK datasets, they share a similar data structure and therefore suitable for our cross-institution experiment.

For all datasets, each medical visit can be represented as the combination of medical codes (i.e., ICD-9 diagnosis codes, CPT procedure codes, and NDC medications). Table 1 lists the detail statistic about the datasets.

**Table 1:** Statistics of the datasets.

Dataset	PFK	Suicide	Asthma	PFK-2013	MIMIC-3
# of patients	1,881,020	79,331	22,862	160,339	7,537
Avg. age	10.3	13.02	8.6	8.8	–
Male %	49.8	49.7	57	50.0	–
# of visits	19,671,825	927,318	432,472	1,880,537	19,993
Avg. # of visits per patient	10.4	11.7	18.9	11.7	2.6
# of unique medical codes	49,835	12,066	8,912	14,190	4,894
Avg. # of medical codes per visit	2.8	2.8	2.8	2.9	13
Max # of medical codes per visit	79	48	43	51	39

### Experimental setup

**Figure 3:**
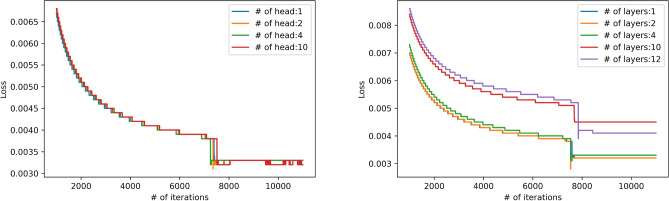
Pre-training analysis with respect to the number of transformer layers and attention head.

We used 80% of the patients as the training set and 20% as the validation set for model pre-training, and 50% of the patients as the training set and 50% as the validation set for population specific tasks. ClaimPT is trained for up to 1000 epochs (i.e., 1000 iterations over the entire training data). The minibatch size was set to 100. To avoid overfitting, we applied the dropout layer, L-2 norm regulation, and early stopping techniques. The size of the all hidden layer was set to 100 to guarantee sufficient expressive power. The number of transformer layers was set to one, while the number of attention heads was set to four. We adjust the number of transformer layers and attention head for training time analysis, as shown in Fig. 3 (the default of layer and head are set to 4 and 2). We can observe that extra transformer layers and attention heads do not benefit the model performance in the pre-training stage. We used RMSprop as the optimizer with a learning rate equal to 0.001. All models were implemented with Python Tensorflow and trained on a server with eight Nvidia Tesla P100 GPUs.

## Results

This section demonstrates the effectiveness of our Claim-PT framework on different downstream population-specific tasks by comparing it with several baselines. First, we conduct two embedding visualization tasks as sanity checks to validate the model’s ability to capture the semantic meaning of patients’ medical codes and health conditions. Next, we evaluate the performance of the proposed framework on two real-world medical predictive tasks using claims data. Specifically, we conduct a suicide prediction task and an asthma exacerbation task. The two cases are different in patient-selection criteria and therefore yield different target populations. The criteria and model performance are presented in detail for each of the tasks.

### Sanity check: embedding visualization

Deep learning models understand the data by projecting the inputs into a latent space, where relevant and salient features for solving the problem at hand are well represented and extracted. In our case, the salient features refer to the semantic meaning of patients’ medical codes and health conditions. Therefore, we first conduct two sanity checks to verify whether our framework can successfully capture the semantic information of medical codes and stratify patients according to their health conditions.

**Figure 4:**
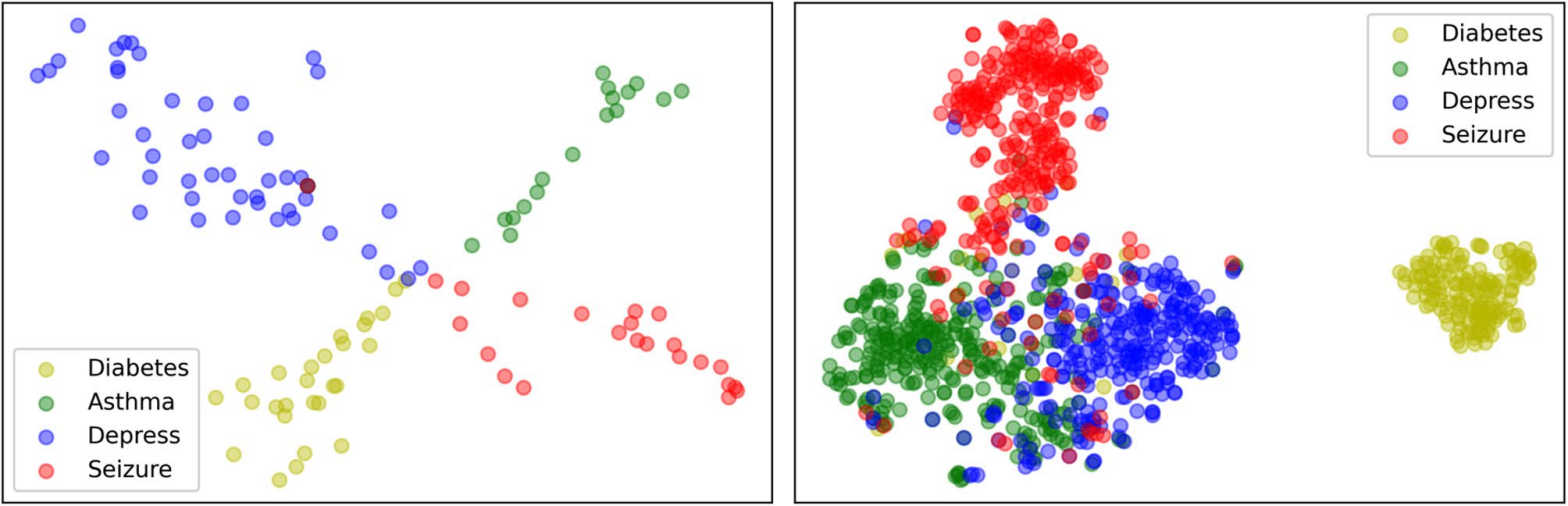
t-SNE scatter plots of diagnosis codes (left) and patients (right) learned by our framework. Four diseases (i.e., diabetes, asthma, depression, and seizure) are selected to provide clear visualization results. ICD-9 diagnosis codes (left) and patients (right) that describe the corresponding disease condition are selected.

We can observe reassuring patterns from the two embedding visualization plots in Fig. 4. For instance, patients with the same disease are grouped together, while patients that are diagnosed with different diseases are well-separated. This observation indicates that our framework is able to capture the clinical meaningful information within claims data and learn efficient embeddings.

### Suicide prediction

Suicide among young people is one of the severe public concerns^33^, which is the second leading cause of death for children according to the American Academy of Child and Adolescent Psychiatry (AACAP). In 2017, about 1 in 13 adolescents have attempted suicide, and these suicide attempts cause a significant healthcare burden. Thus, it is critical in clinical practice to predict the risk of suicide accurately.

Recently, efforts have been made to apply the machine learning architecture to model the sequential claims data to predict suicide risk^13^. However, the excluding criteria limited the patient population (i.e., only a couple of hundreds of patients have committed suicide in one institution’s database) and therefore restricted the model performance due to inadequately training.

**Figure 5:**
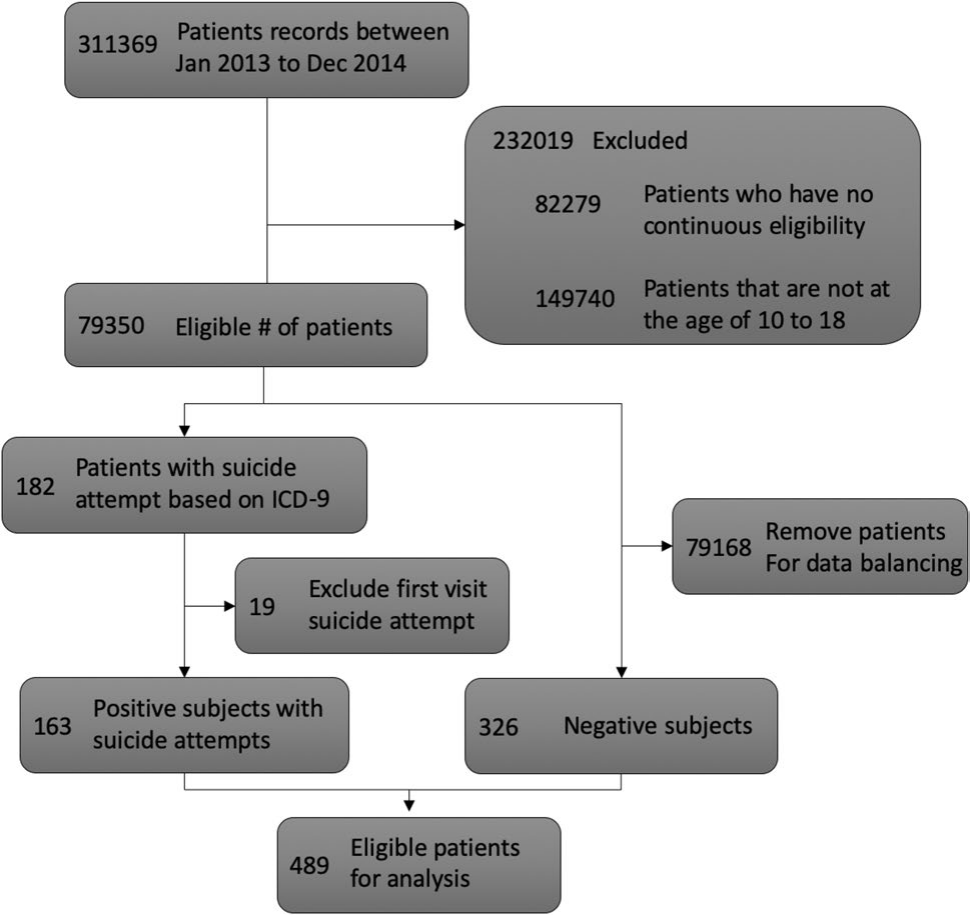
The cohort selection process for the study of suicide risk prediction.

This case study examines whether the excluded patient population can help pre-train the deep learning framework and ultimately provide a model performance boost on suicide risk prediction tasks. In particular, we first construct the patient cohort according to the patient selection criteria shown in Fig. 5. 163 patients who have suicide attempts during 2013–2014 are selected as positive subjects, while 326 patients who do not have suicide attempts in the same time span are randomly selected as negative subjects. Next, we develop models with the next-visit prediction window, i.e., the model aims to predict the occurrence of a future suicide attempt that happens in the next encounter. Therefore, the model is trained using the patient’s medical visits prior to the first suicide attemp (for positive subjects) or the last recorded medical visit (for negative subjects). All Variables that have been used in the pre-training phase are selected for the suicide prediction task. Finally, we randomly divide the patient cohort into 70% training and 30% testing sets.

All models are trained for twenty epochs to ensure sufficient training. The same experiments are repeated five times, and the average model performances are reported for comparison. We leverage Area Under the receiver operator characteristic Curve (AUC), a widely used evaluation metric of discrimination performance that ranges from 0.5 (random prediction) to 1.0 (perfect prediction), to evaluate the predictive performance for all models. The data preprocessing details can be found on our GitHub page.

From Table 2, we can see that the AUC of the proposed pre-training and fine-tuning framework is higher than that of baselines on the suicide risk prediction task. The Claim-PT variants, Claim-PT Start and Claim-PT Pool, use the pre-trained framework for risk prediction without fine-tuning, and the predicted results achieve similar performance compared to baselines. This indicates that our proposed pre-training objectives is able to capture the semantic meaning of medical codes, and thus the framework can distinguish patients based on their health records. Since the suicide risk prediction task contains less than 500 patients, deep learning models, including Doctor AI, Dipole, and TransE, can not gain sufficient predictive power and thus yield suboptimal performance compared to linear regression. We can also observe that the model performance between deep learning models is similar. The reason is likely because the number of patient records and the categories of medical codes is limited, and developing more complicated models with more parameters does not help to sufficiently train the parameters.

**Table 2:** Results on suicide risk prediction task.

	Model	AUC
	LR Sparse^34^	0.73
	LR Dense^34^	0.74
Baselines	DoctorAI^35^	0.72
	Dipole^36^	0.72
	TransE^37^	0.73
Our Model	Claim-PT + Fine-tune	0.84

### Asthma exacerbation

We next turn our attention to the asthma exacerbation prediction task. Asthma is a common yet serious health problem among children, costing billions of dollars every year and imposes a significant burden on the healthcare system in the U.S.^38^. Asthma exacerbation is a severe asthma condition and requires medical interventions, resulting in an emergency department visit or hospitalization, which is resource-consuming and may even result in death. Therefore, it is practically essential to accurately identify asthma exacerbation in advance for better care delivery and care intervention.

**Figure 6:**
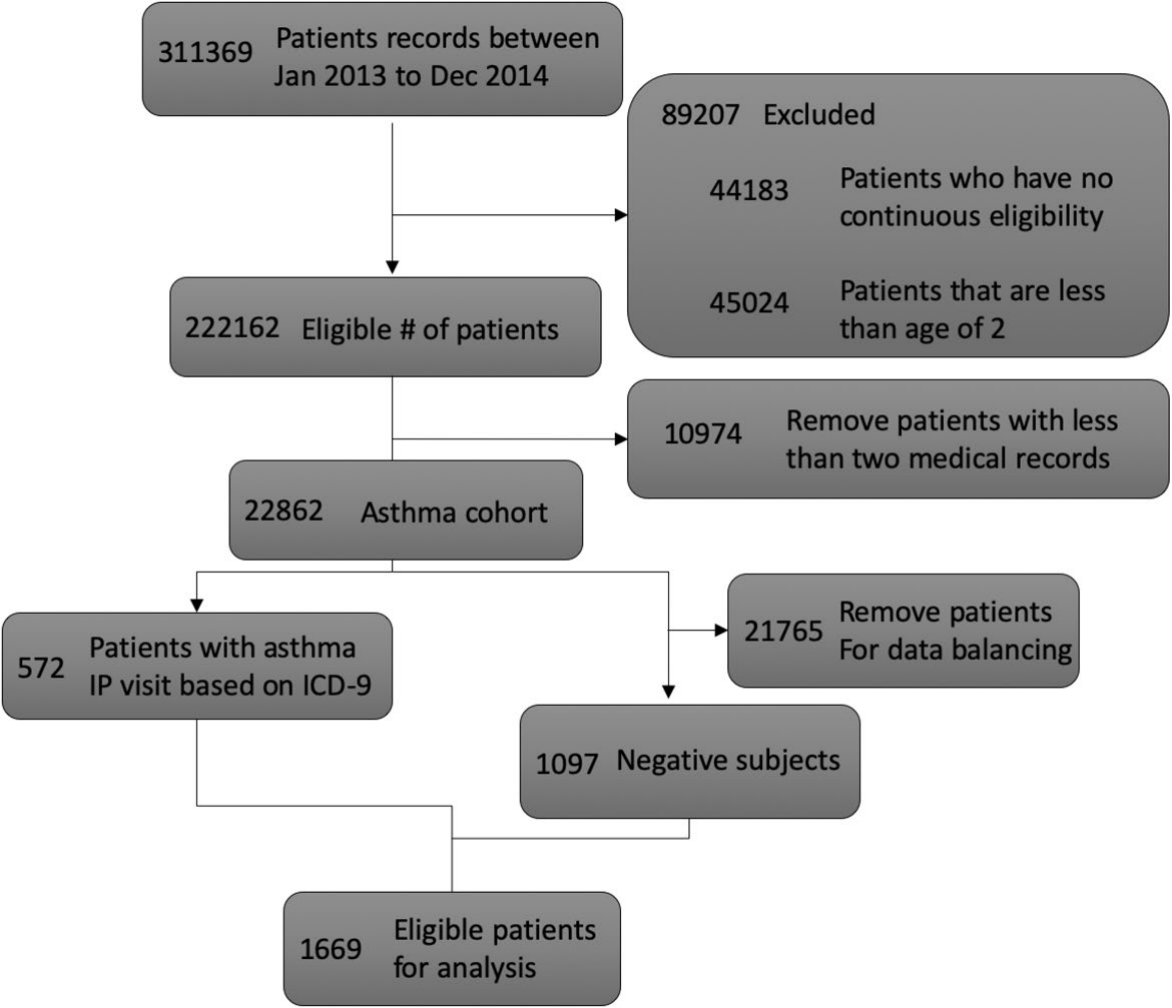
The cohort selection process for the study of asthma exacerbation.

A data-driven study to predict asthma exacerbation using claims data is conducted, and various methods are compared with our Claim-PT framework to illustrate its effectiveness. We adopt the cohort selection process from a recent asthma exacerbation prediction study^29^, and the excluding criteria is shown in Fig. 6. As shown in the figure, 572 patients with in-patient asthma visits (i.e., patients admitted to hospital due to asthma diagnosis) from 2013 to 2014 are identified as the positive subjects, while 1097 randomly selected patients without asthma hospitalization are selected as negative subjects. A total of 1669 patients are identified and form the final cohort. The experiment setup is the same as the suicide risk prediction task, and the detailed preprocessing steps are on our GitHub page.

As shown in Table 3, Our proposed framework showed supervisor model performance compared to all baselines. The AUC of LR Sparse is the highest among all baselines, including the two Claim-PT variants. This observation is likely because the prediction of asthma exacerbation depends on the frequency of specific medical codes, such as the previous hospitalization. LR Sparse captures relationships between these medical codes and the prediction outcome, while LR Dense loses granularity information through feature preprocessing steps. Although RNN-based models, Doctor-AI and Dipole, are able to capture the sequential information, they have a complex model structure and thus yield suboptimal performance. The inferior performance of TransE can confirm this surmise. TransE utilizes the state-of-the-art transformer block as encoder and has more parameters, but the AUC score is the lowest among all approaches.

**Table 3:** Results on asthma exacerbation prediction task.

	Model	AUC
	LR Sparse^34^	0.77
	LR Dense^34^	0.73
Baselines	DoctorAI^35^	0.74
	Dipole^36^	0.73
	TransE^37^	0.71
Our Model	Claim-PT + Fine-tune	0.82

### Ablation experiments

In this subsection, an ablation study is conducted to validate the effectiveness of the framework and explore critical clinical information with respect to the predictive results. Specifically, we first compared two zero-shot models with the fine-tuned model to illustrate the necessity and advantages of the fine-tuning stage. Next, we explain the performance of the proposed model by comparing it with the reduced models that only leverage partial input information.

**Figure 7:**
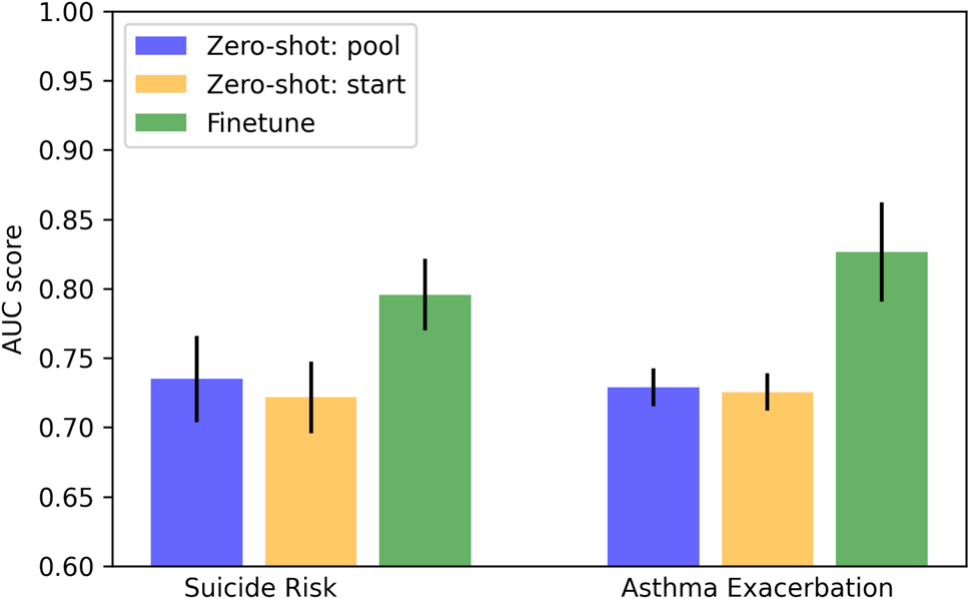
Effectiveness of the pre-training stage.

**Figure 8:**
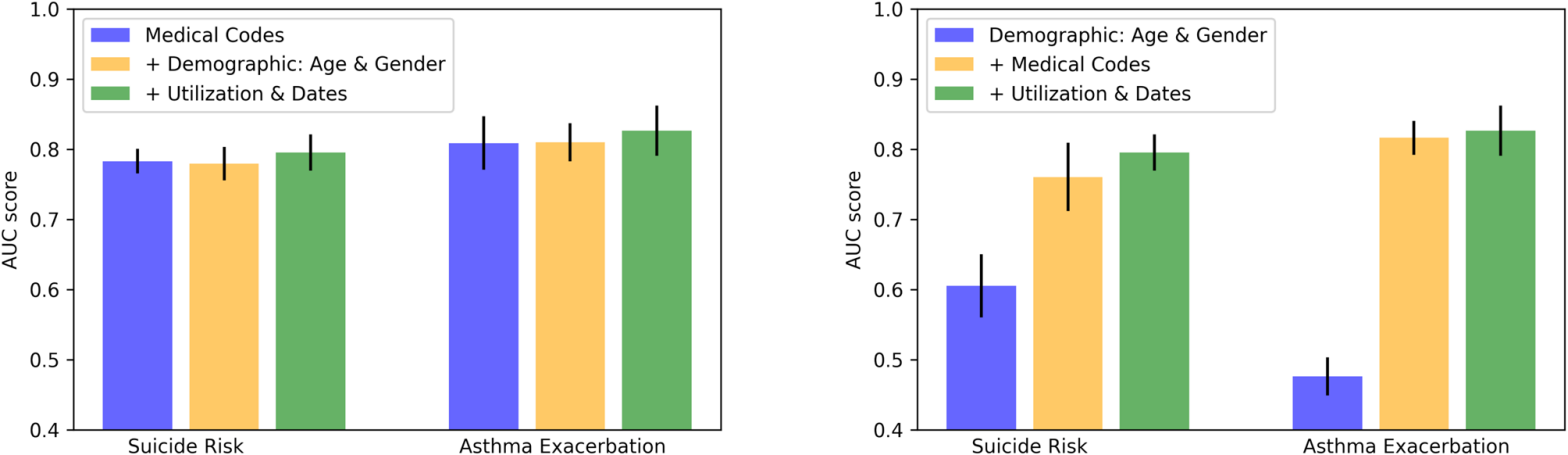
Effectiveness of adding different input information from different order.

Figures  7 and  8 show the experimental results under AUC evaluation metrics for predicting suicide risk and asthma exacerbation, where a higher AUC score indicates better model performance. From Fig. 7, we can observe that our framework showed superior model performance after fine-tuning compared to the two zero-shot approaches. This observation is expected as the framework focused on optimizing the two general objectives (i.e., NVP and CP) at the pre-training stage. Compared to the zero-shot start method, the zero-shot pool shown higher AUC scores in both tasks. The better model performance is likely because the pooling operation can extract the salient features within the input sequences. We can also confirm that the framework is able to capture clinical-meaningful information at the pre-training stage as the performances of two zero-shot approaches are better than random guesses.

In Fig. 8, the model performance with different input variables is shown. We can observe that the pre-training model can gain sufficient predictive power on the two downstream predictive tasks, adding demographic and utilization information can barely improve the model performance. This observation is consistent with experimental results found in the previously study^30^. The marginal improvement is likely due to the fact that medical codes often contain information that is overlapped with medical utilization information and demographic information. From Fig. 8 (right), we can see that only using demographic information will result in poor model performance in both tasks, especially in predicting asthma exacerbation. This observation matches our intuition as the suicide risk is more likely to correlate with age and gender, while asthma exacerbation probability is less likely associated with age and gender. It is also interesting to observe that adding medical information can significantly boost the model performance while adding utilization information improves marginally. Such an improvement pattern is likely because the medical codes sometimes carry utilization information.

**Figure 9:**
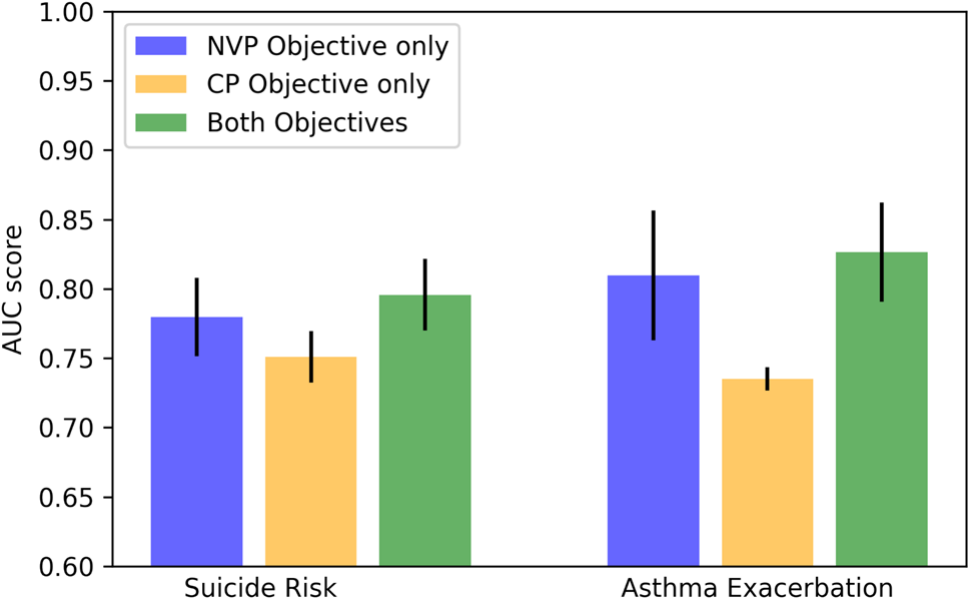
Effectiveness of different training objectives.

Figure 9 helps us understand the model performance concerning the two proposed training objectives (i.e., NVP and CP). We can observe that model with both training objectives shown the highest AUC score. This indicates that both objectives are beneficial to learning efficient patient representation and helps predictive downstream tasks. In addition, we can see that using the NVP training objective only can yield good performance. This is likely because NVP enables our model to encode the clinical meaning of medical codes by capturing their co-occurrence information. The learned clinical meaning might overlap with the domain expert’s knowledge (i.e., the medical grouper) and, therefore, only marginally improves the model performance when adding the CP objective.

### Knowledge transfer across institutes

As we observed from the above experiments, the performance of the deep learning model on clinical events is highly dependent on the scale of the dataset. However, many institutions have not yet collected large-scale datasets or can not access sufficient amounts of data due to privacy issues. In these cases, training deep learning from scratch will result in suboptimal model performance and could easily lead to overfitting. One possible way to mitigate this challenge is to transfer knowledge from an institute with a large number of patient records. In this subsection, we evaluate the ability of our framework to transfer knowledge across institutes.

**Figure 10:**
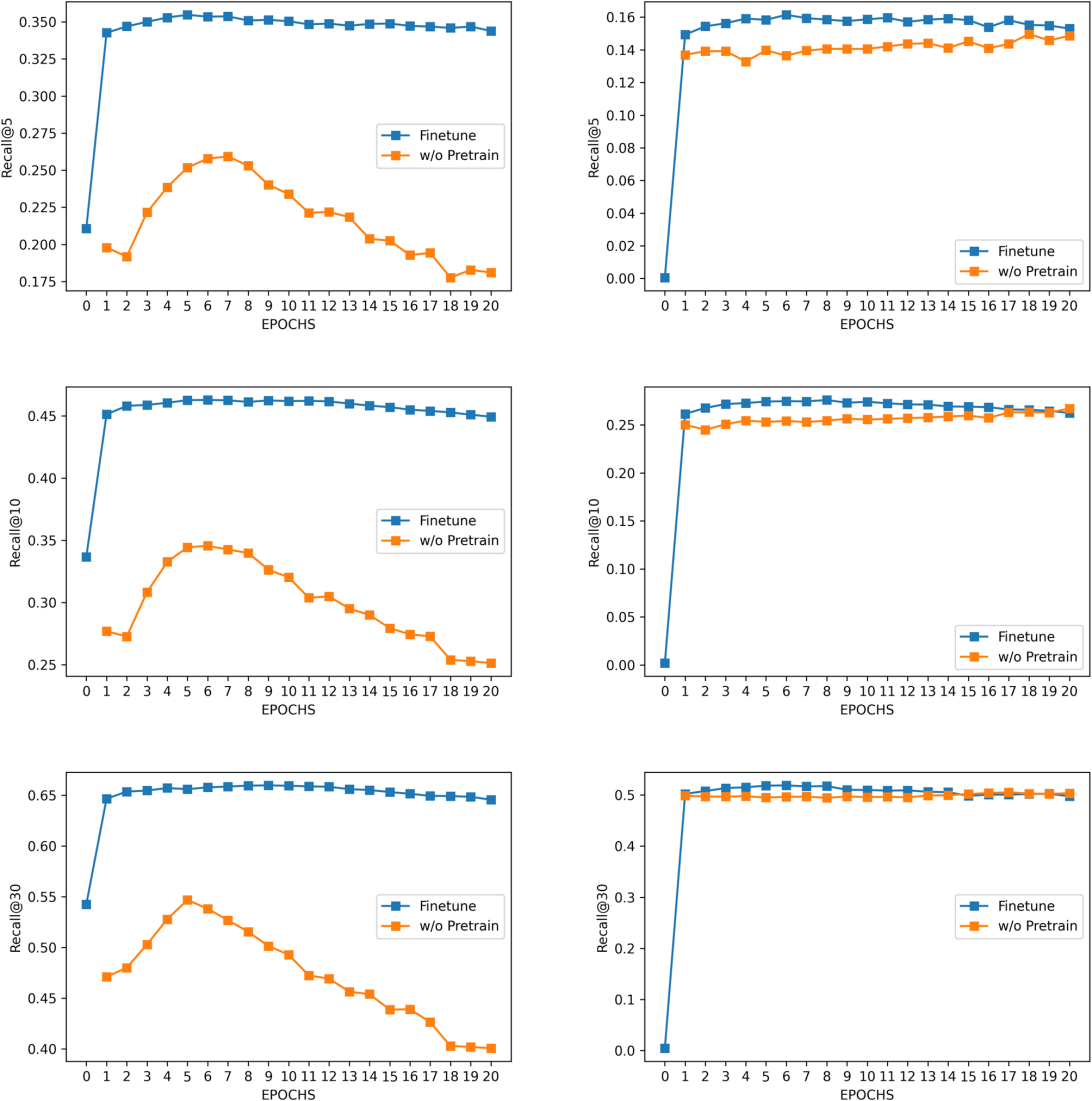
The impact of pre-training on improving the performance on PFK-2013 (left) and MIMIC-3 (right), PFK-2013 is a subset of the PFK pediatric claims dataset (not used in the pre-training phase), while MIMIC-3 contains ICU patient records. The pre-training procedure results in less than 5% in the model performance for MIMIC-3, but more than 10% improvement for PFK-2013.

Figure 10 demonstrates the experiments on PFK-2013 and MIMIC-3. In Fig. 10 (left), we can observe a vast improvement of the prediction performance induced by knowledge transfer from pre-training. This improvement is because PFK-2013 shares a similar data distribution as PFK (i.e., they both contain pediatric patient records in the Ohio area), enabling the knowledge to transfer from a large dataset to a small dataset successfully. Due to privacy limitations, we can not access another pediatric claims dataset, but we believe the impact of pre-training on improving model performance is still valid.

In Fig. 10 (right), we test our pre-trained framework on the MIMIC-3 dataset to explore the cross-domain adaptation result. As seen in the figure, the improvement is barely noticeable. The observation matches the clinical intuition as the MIMIC-3 and PFK have a significantly different patient population, where the former contains elderly and severely ill patients in the ICU department and the latter contains children that often visit the outpatient department.

## Limitation

This study has several limitations. First of all, the findings and conclusions were made based on the experimental results on a state Medicaid pediatric claims dataset. The results and observations may vary by state, insurer plan, population age distribution, or payer type. Second, we conducted the study under specific data preprocessing rules, such as continuous enrollment eligibility. These enforcements may have distorted the underlying population. Third, this study focuses on claims data only. Compared to EHR data, claims data do not contain important clinical information such as vital signs, medical notes, and visual images. Lastly, the black-box nature of the proposed deep learning framework hinders the users or physicians from interpreting the predictive results.

Each of the limitations mentioned above points to a good direction. Our future work will try to address these limitations. We plan to incorporate with other healthcare institutes to extend our study scope with more comprehensive datasets. In addition, we will try to collaborate with physicians to deploy the model in the care organization to provide better guidance for care management. Finally, we are considering leveraging the bootstrapping method and sequential attention mechanism to provide confidence interval estimation and consistent interpretation of the model predictions.

## Conclusion

This study proposed Claim Pre-Training (Claim-PT), a task-agnostic pre-training and fine-tuning framework that aims to achieve solid clinical concepts understanding and efficient patient representation learning. By pre-training the transformer-based model on large pediatric claims data with millions of patient records, our model acquires significant medical knowledge, understanding, and ability to process unevenly distributed longitudinal medical claims. The sufficiently pre-trained model can then be leveraged to solve downstream population-specific tasks, which often have a small patient cohort and therefore limit the performance of deep learning models. We conducted three experiments to validate the effectiveness of our Claim-PT framework, i.e., suicide risk prediction task, asthma exacerbation prediction, and auto diagnosis task. The experimental results suggest that our Claim-PT framework can significantly boost model performance on discriminative tasks, achieving more than 10% performance gain. We also demonstrate that Claim-PT has the potential to transfer knowledge from one institution to another institution. In summary, our study investigates the possibility of using unsupervised learning to learn general medical knowledge from claims data. The promising results imply that our pre-train and fine-tune framework can be beneficial in solving downstream population-specific tasks.

## Data Availability

The data source for this study is claims data from Partner For Kids (PFK). Partner for Kids (PFK) is one of the largest pediatric ACOs for Medicaid enrollees in central and southeastern Ohio. The dataset was obtained from a density sampled study that contains more than 600,000 enrollees’ medical claims. In accordance with the Common Rule (45 CFR 46.102[f]) and the policies of Nationwide Children’s Institutional Review Board, this study used a limited dataset and was not considered human subjects research and thus not subject to institutional review board approval. Restrictions apply to the availability of these data, and so are not publicly available. More information can be found: https://partnersforkids.org/. For help getting access to the dataset, please contact: PartnersForKids@NationwideChildrens.org.

## References

[1] Choi E, Schuetz A, Stewart WF, Sun J (2017). Using recurrent neural network models for early detection of heart failure onset. J. Am. Med. Inform. Assoc..

[2] Landi I (2020). Deep representation learning of electronic health records to unlock patient stratification at scale. NPJ Digit. Med..

[3] Zeng, X. *et al.* Multilevel self-attention model and its use on medical risk prediction. In *Pacific Symposium on Biocomputing 2020*, 115–126 (World Scientific, 2019). 31797591

[4] Sun, C., Shrivastava, A., Singh, S. & Gupta, A. Revisiting unreasonable effectiveness of data in deep learning era. In *Proceedings of the IEEE International Conference on Computer Vision*, 843–852 (2017).

[5] Hedderich, M. A. & Klakow, D. Training a neural network in a low-resource setting on automatically annotated noisy data. arXiv:1807.00745 (arXiv preprint) (2018).

[6] Haines-Delmont A (2020). Testing suicide risk prediction algorithms using phone measurements with patients in acute mental health settings: Feasibility study. JMIR mHealth uHealth.

[7] Choi, E. *et al.* Generating multi-label discrete patient records using generative adversarial networks. In *Machine Learning for Healthcare Conference*, 286–305 (PMLR, 2017).

[8] Helgheim BI, Maia R, Ferreira JC, Martins AL (2019). Merging data diversity of clinical medical records to improve effectiveness. Int. J. Environ. Res. Public Health.

[9] Seneviratne, M. G., Kahn, M. G. & Hernandez-Boussard, T. Merging heterogeneous clinical data to enable knowledge discovery. In *Biocomputing 2019: Proceedings of the Pacific Symposium*, 439–443 (World Scientific, 2018). PMC6447393 30864344

[10] Lee D (2020). Generating sequential electronic health records using dual adversarial autoencoder. J. Am. Med. Inform. Assoc..

[11] Buczak AL, Babin S, Moniz L (2010). Data-driven approach for creating synthetic electronic medical records. BMC Med. Inform. Decis. Mak..

[12] Ma, F. *et al.* Risk prediction on electronic health records with prior medical knowledge. In *Proceedings of the 24th ACM SIGKDD International Conference on Knowledge Discovery and Data Mining*, 1910–1919 (2018).

[13] Su, K.-Y., Su, J., Wiebe, J. & Li, H. Proceedings of the joint conference of the 47th annual meeting of the ACL and the 4th international joint conference on natural language processing of the afnlp. In *Proceedings of the Joint Conference of the 47th Annual Meeting of the ACL and the 4th International Joint Conference on Natural Language Processing of the AFNLP* (2009).

[14] Peters, M. E. *et al.* Deep contextualized word representations. arXiv:1802.05365 (arXiv preprint) (2018).

[15] Baevski, A., Edunov, S., Liu, Y., Zettlemoyer, L. & Auli, M. Cloze-driven pretraining of self-attention networks. arXiv:1903.07785 (arXiv preprint) (2019).

[16] Peters, M. E., Ammar, W., Bhagavatula, C. & Power, R. Semi-supervised sequence tagging with bidirectional language models. arXiv:1705.00108 (arXiv preprint) (2017).

[17] Guo, Y. *et al.* Spottune: transfer learning through adaptive fine-tuning. In *Proceedings of the IEEE/CVF Conference on Computer Vision and Pattern Recognition*, 4805–4814 (2019).

[18] Kan M, Wu J, Shan S, Chen X (2014). Domain adaptation for face recognition: Targetize source domain bridged by common subspace. Int. J. Comput. Vision.

[19] Shao L, Zhu F, Li X (2014). Transfer learning for visual categorization: A survey. IEEE Trans. Neural Netw. Learn. Syst..

[20] Li Y (2020). Behrt: Transformer for electronic health records. Sci. Rep..

[21] Lee J (2020). Biobert: A pre-trained biomedical language representation model for biomedical text mining. Bioinformatics.

[22] Alsentzer, E. *et al.* Publicly available clinical bert embeddings. arXiv:1904.03323 (arXiv preprint) (2019).

[23] Devlin, J., Chang, M.-W., Lee, K. & Toutanova, K. Bert: Pre-training of deep bidirectional transformers for language understanding. arXiv:1810.04805 (arXiv preprint) (2018).

[24] Mikolov, T., Chen, K., Corrado, G. & Dean, J. Efficient estimation of word representations in vector space. arXiv:1301.3781 (arXiv preprint) (2013).

[25] He, K., Girshick, R. & Dollár, P. Rethinking imagenet pre-training. In *Proceedings of the IEEE/CVF International Conference on Computer Vision*, 4918–4927 (2019).

[26] Rasmy L, Xiang Y, Xie Z, Tao C, Zhi D (2021). Med-bert: Pretrained contextualized embeddings on large-scale structured electronic health records for disease prediction. NPJ Digit. Med..

[27] Choi, E. *et al.* Multi-layer representation learning for medical concepts. In *Proceedings of the 22nd ACM SIGKDD International Conference on Knowledge Discovery and Data Mining*, 1495–1504 (2016).

[28] Su C (2020). Machine learning for suicide risk prediction in children and adolescents with electronic health records. Transl. Psychiatry.

[29] Xiang Y (2019). Asthma exacerbation prediction and interpretation based on time-sensitive attentive neural network: A retrospective cohort study. medRxiv.

[30] Zeng X, Lin S, Liu C (2021). Multi-view deep learning framework for predicting patient expenditure in healthcare. IEEE Open J. Comput. Soc..

[31] Choi, E. *et al.* Retain: An interpretable predictive model for healthcare using reverse time attention mechanism. arXiv:1608.05745 (arXiv preprint) (2016).

[32] Ma, F. *et al.* Kame: Knowledge-based attention model for diagnosis prediction in healthcare. In *Proceedings of the 27th ACM International Conference on Information and Knowledge Management*, 743–752 (2018).

[33] Nock MK (2013). Prevalence, correlates, and treatment of lifetime suicidal behavior among adolescents: Results from the national comorbidity survey replication adolescent supplement. JAMA Psychiatry.

[34] Weisberg S (2005). Applied Linear Regression.

[35] Choi, E., Bahadori, M. T., Schuetz, A., Stewart, W. F. & Sun, J. Doctor AI: Predicting clinical events via recurrent neural networks. In *Machine Learning for Healthcare Conference*, 301–318 (PMLR, 2016). PMC5341604 28286600

[36] Ma, F. *et al.* Dipole: Diagnosis prediction in healthcare via attention-based bidirectional recurrent neural networks. In *Proceedings of the 23rd ACM SIGKDD International Conference on Knowledge Discovery and Data Mining*, 1903–1911 (2017).

[37] Yüksel, A. E., Türkmen, Y. A., Özgür, A. & Altınel, B. Turkish tweet classification with transformer encoder. In *Proceedings of the International Conference on Recent Advances in Natural Language Processing (RANLP 2019)*, 1380–1387 (2019).

[38] Nurmagambetov T, Kuwahara R, Garbe P (2018). The economic burden of asthma in the united states, 2008–2013. Ann. Am. Thorac. Soc..

